# Deterministic and Probabilistic Risk Assessment of Chlorpyrifos Residues via Consumption of Tomato and Cucumber in Armenia

**DOI:** 10.3390/foods14162871

**Published:** 2025-08-19

**Authors:** Meline Beglaryan, Taron Kareyan, Monika Khachatryan, Bagrat Harutyunyan, Davit Pipoyan

**Affiliations:** Center for Ecological-Noosphere Studies, NAS RA, Yerevan 0025, Armenia; meline.beglaryan@cens.am (M.B.); taron.kareyan@cens.am (T.K.); monika.khachatryan@cens.am (M.K.); bagrat.harutyunyan@cens.am (B.H.)

**Keywords:** pesticide residue, food safety, dietary exposure, risk

## Abstract

Chlorpyrifos (CPF) is a widely used organophosphate insecticide; however, global concerns exist regarding its potential health risks, particularly developmental neurotoxicity. This study aimed to determine CPF residues in locally sourced tomatoes and cucumbers and assess the potential chronic and acute dietary risks associated with their consumption by the adult population of Armenia. As part of the national residue monitoring program, samples of the two most commonly consumed vegetables (tomato and cucumber) were collected from various regions of Armenia and analyzed using gas chromatography–tandem mass spectrometry (GC-MS/MS). Two databases were used for dietary exposure assessment: one containing CPF residue levels and another containing individual food consumption data from a food frequency questionnaire completed by 1329 Armenian residents. Chronic risk was assessed using the Margin of Exposure (MOE), while acute risk was evaluated using the Hazard Quotient (HQ) and the Hazard Index (HI). CPF residues were detected in 15% of tomato and 28.6% of cucumber samples, with a mean content of 0.003 mg/kg. Deterministic and probabilistic assessments indicated no health concern (i.e., MOE > 300 and >1000, HQ and HI < 1) for the general adult population at current exposure levels. However, higher cumulative risk estimates obtained for high-consumption groups emphasize the significance of these studied vegetables as notable contributors to overall CPF intake. The findings indicate the importance of establishing vegetable-specific maximum residue levels, strengthening monitoring, and considering vulnerable population groups in future research. Broader assessments, including other plant-origin products, are recommended to ensure comprehensive risk assessment and support science-based policy decisions for improved food safety and public health protection in Armenia.

## 1. Introduction

Pesticides are one of the major environmental contaminants, with a wide variety of types used globally [1]. Among them, chlorpyrifos (CPF) is a commonly applied organophosphate insecticide known for its multi-target effectiveness, particularly in agricultural pest management [2]. Despite its contribution to pest control and improved crop yields, the use of CPF raised significant environmental and public health concerns [3,4]. CPF can persist in the environment, accumulate in living organisms, and enter the food chain [5,6]. Notably, CPF was reported to be among the most frequently detected pesticides globally [7].

CPF has demonstrated toxicity to non-target organisms, including humans, even at low concentrations [8]. It is categorized as second-class moderately hazardous to humans, based on the WHO/IPCS classification [9]. Toxicological studies have linked CPF exposure to adverse health effects, associating its toxicity with several physiological disorders including neurological, endocrine disruption (ED), ovarian cancer, and hematological and reproductive abnormalities. One of the key toxicological endpoints of CPF exposure is acetylcholinesterase inhibition, along with its neurodevelopmental effects [10,11,12]. Occupationally exposed populations, such as farmers and pesticide applicators, are especially vulnerable, but risks through dietary intake also exist for the general population [11]. Over decades, the acceptable daily intake (ADI) of CPF has been reduced to 0–0.01 mg/kg body weight [13,14]. According to the European Food Safety Authority (EFSA) statement, chlorpyrifos and chlorpyrifos-methyl can present a potential risk to human health, particularly related to the possible genotoxic effects and developmental neurotoxicity (DNT). Also, due to insufficient data for establishing safe exposure levels, the approval criteria for human health set by EU legislation were not met [15]. In response to these scientific concerns, regulatory authorities have taken steps to restrict or eliminate the use of CPF. In early 2020, the European Commission confirmed the decision not to renew the authorization for the insecticides chlorpyrifos and chlorpyrifos-methyl [16]. In addition, EU Member States supported the Commission’s proposal to reduce the maximum residue levels (MRLs) of these pesticides to the minimum levels that can be detectable by laboratory analysis [17,18]. Similarly, the US Environmental Protection Agency (EPA) withdrew all previous tolerances for CPF and is engaged with product registration to further reduce exposure associated with the remaining authorized uses of CPF [19,20]. In 2024, there was a proposal to list CPF as a persistent organic pollutant (POP) under the Stockholm Convention, having met all criteria justifying the need for global action [21].

Nevertheless, regulatory bans and restrictions in many countries have not prevented the use and occurrence of CPF in others [22], including Armenia. Previously, national studies have documented widespread use of pesticides in agriculture, with organophosphates accounting for approximately 20% of total pesticide usage. A total of 75.1% of surveyed respondents mentioned that they routinely apply pesticides on an annual basis, whereas an additional 7.7% reported occasional usage, resulting in 82.8% being identified as pesticide users. Among these, insecticides were the most frequently applied [23]. However, data on the occurrence of the insecticide CPF in Armenian food products remains limited, and no prior studies have investigated CPF residues in foods produced or commonly consumed in Armenia, nor has any dietary risk assessment been conducted for these products.

Tomato (*Solanum lycopersicum*) and cucumber (*Cucumis sativus*) are among the most extensively cultivated and consumed vegetables both globally and within Armenia. These crops are commonly treated with insecticides, including organophosphates such as CPF. The chemical stability of CPF and its resistance to degradation during usual food preparation make it particularly difficult to eliminate. For example, studies indicated that unpeeled vegetables like tomatoes and cucumbers tend to retain CPF residues, which can penetrate from the surface to the outer layers. Although washing, peeling and food preparation (e.g., pickling) can reduce residual levels, these measures may not fully remove the pesticide, especially for raw-consumed vegetables [24,25]. Consequently, dietary exposure through such vegetables may present a higher risk.

In Armenia, tomato and cucumber hold significant agricultural and dietary value. According to the 2023 National Food Balance of the Republic of Armenia, tomato production reached 158.6 thousand tons and cucumber production 69.4 thousand tons. The country is nearly self-sufficient in cucumber production (95%) and produces a surplus of tomatoes (106.7%). The annual per capita consumption is also notable high, with an average of 50.8 kg of tomato and 20.3 kg of cucumber consumed per person [26]. These figures reflect the importance of these two vegetables in the Armenian diet and the critical need to ensure their safety.

Considering the global concerns regarding the occurrence and toxicity of CPF, and the existing data gap in Armenia, this study represents the first national effort to assess CPF residues specifically in locally produced and consumed tomatoes and cucumbers. Therefore, the study aimed to quantify CPF residues in these vegetables and to assess both chronic and acute dietary exposure to the Armenian adult population. This is the first probabilistic dietary risk assessment for CPF in commonly consumed vegetables (tomatoes and cucumbers) produced in Armenia.

## 2. Materials and Methods

### 2.1. Sampling and Analyses

Fresh tomato and cucumber samples were collected from various regions (also known as marzes) of Armenia in 2019, as part of the National Residue Monitoring Program for pesticides, nitrates, heavy metals, and genetically modified organisms in plant origin products. Sampling was conducted directly from the comparatively large agricultural plots in different regions of Armenia that are among the key suppliers of locally sourced tomatoes and cucumbers to markets across all regions of the country. Following the standard operational procedures (SOPs) developed by the Center for Ecological-Noopshere Studies (CENS) [27], several subsamples of tomatoes and cucumbers were randomly collected from each agricultural plot. Sampling was conducted across the entire plot, excluding about 1 m from the plot edges and, in the case of furrows, about 1 m from the end. The randomly collected subsamples were then combined to form one composite, representative sample for each vegetable from each agricultural plot in the studied regions of Armenia. Each composite sample was prepared from at least 12 subsamples taken from the agricultural plot to ensure the representativeness of the primary production sources.

In total, 20 tomato and 14 cucumber composite samples were prepared and analyzed for the presence of CPF using gas chromatography–tandem mass spectrometry (GC-MS/MS, TSQ 8000 Thermo Fisher Scientific, Waltham, MA, USA), with a limit of quantification (LOQ) of 0.001 mg/kg and limit of detection (LOD) of 0.0003 mg/kg. A 10 g portion of each vegetable sample was prepared using the QuEChERS (Fast, Easy, Inexpensive, Effective, Robust and Safe) [28] procedure and analyzed in the laboratory of the Republican Veterinary-Sanitary and Plant-Sanitary Laboratory Services Center (RVSPCLS) SNCO, which is internationally accredited in accordance with the ISO 17025 standard. As part of the sample pretreatment, 1 mL of sample extracts was reconstituted using acetone/hexane 1:1 after evaporation of the acetonitrile extraction solvent. The extract was then analyzed using a GC-MS/MS system equipped with a fast GC column for high-throughput separation. Quality control (QC) procedures included the analysis of procedural blanks, spiked samples, and replicates to ensure accuracy and precision throughout the investigation.

### 2.2. Dietary Exposure Assessment and Risk Characterization

To assess both chronic and acute dietary exposure to CPF due to the consumption of locally sourced tomatoes and cucumbers by the adult population in Armenia, the estimated daily intake (EDI) was calculated using the following equation:

(1) $$EDI=\frac{{C_{content}\times C}_{food}}{BW}$$

where C_content_ is the average CPF residue level in each studied vegetable, C_food_ represents the consumption data for each vegetable (kg/day), and BW is the average body weight for the adult population in Armenia (71.5 kg).

For EDI calculations, the detected average residue levels (C_content_) of CPF were used. However, for “left-censored” data (i.e., samples in which CPF residues were below the LOD), different approaches were considered [29]—values below the LOD were assumed to be equal to 0 (lower-bound (LB) approach), LOD/2 (middle-bound (MB) approach), or equal to the LOD (upper-bound (UB) approach)—to understand whether these assumptions affect the overall interpretation of the study results when using detected average residue levels of CPF. It is important to note that, when considering the LB, MB, and UB scenarios, the initial calculations showed only minor differences in numerical results. Therefore, both deterministic and probabilistic calculations for dietary exposure and the values presented in this study are based on the average detected CPF residue levels. Individual-based consumption data (C_food_) for tomatoes and cucumbers were extracted from a food frequency questionnaire (FFQ) survey, conducted anonymously and face-to-face among the representative sample of the adult population (1329 residents aged 18 and older, including 563 males and 766 females) in the capital city of Yerevan, Armenia, by the Informational-Analytical Center for Risk Assessment of the Food Chain at CENS.

The FFQ included questions on food consumption frequencies (ranging from “no consumption” to “2–4 times per week”, as well as less frequent categories such as “2–3 times per month”, “once a month”, and “other”) and an open-ended question on portion sizes. It also collected information on food sources and demographic characteristics, including gender, age, education, employment, and income level. Data analyses were conducted using IBM SPSS Statistics (version 22.0), with particular application of the “Visual Binning” feature to categorize consumption levels and consumer groups accordingly. Chronic EDI values were calculated using average daily consumption data, whereas acute EDI was based on consumed portions of tomatoes and cucumbers among the identified consumer groups. This approach allowed us to consider all possible scenarios for CPF exposure assessments.

To assess and characterize the potential risks associated with chronic dietary exposure to CPF in each studied vegetable, the Margin of Exposure (MOE) was calculated using the following equation [14]:

(2) $$MOE=\frac{HBGV}{EDI}$$

where HBGV (mg/kg bw/day) represents the health-based guidance value for CPF, as available in the literature. EFSA has stated that, in the absence of established toxicological reference values, it is not possible to perform a reliable risk assessment for consumers. This presents a major concern in the case of CPF. However, to enable chronic risk assessment in the present study, an existing toxicological reference value like the HBGV was used. Therefore, the chronic MOE was calculated considering the developmental neurotoxicity lowest observed adverse effect level (DNT LOAEL) of 0.3 mg/kg bw/day as defined by EFSA [15].

In the context of cumulative chronic risk assessment, based on the combined consumption of two studied vegetables (tomato and cucumber), the ∑MOE was calculated by dividing DNT LOAEL by the ∑EDI. ∑EDI is the sum of the EDI values of CPF from both tomato and cucumber consumption. For chronic risk characterization, the approach mentioned by Tarazona et al. [14] was considered, according to which MOE values below 300 indicate a potential health concern, and MOE values between 300 and 1000 may also raise concerns.

To evaluate and characterize the potential risks from acute dietary exposure to CPF through the consumption of each studied vegetable, the Hazard Quotient (HQ) was calculated using the following equation [30]:

(3) $$HQ=\frac{EDI}{ARfD}$$

where ARfD (mg/kg bw/day) is the acute reference dose. van der Voet et al. [30] reported that to provide a clear indication of current exposure of previously authorized pesticide active substances like CPF, an artificially low ARfD of 0.0001 mg/kg bw/day was adopted in 2020. This artificial value is intended to trigger exceedance alerts if CPF is detected in food. Moreover, the earlier valid ARfD of 0.005 mg/kg bw/day established in 2014 [30] was also considered; however, the data presented in the study correspond to the lowest ARfD of 0.0001 mg/kg bw/day.

For cumulative acute risk assessment in the case of the combined consumption of two vegetables, the Hazard Index (HI) was calculated by summing the Hazard Quotient (HQ) values obtained for CPF in tomato and cucumber. According to the report by van der Voet et al. [30], the calculated HQ and HI values for CPF were compared to the reference value of 1. Values below 1 indicated no health concern related to CPF exposure.

All dietary risk assessments in the present study (MOE, HQ and HI) were conducted using both deterministic (point estimate) and probabilistic approaches. Deterministic assessment provides single-value estimates based on fixed input data, while probabilistic assessments account for variability and uncertainty by considering a range of possible values. In the probabilistic approach, instead of calculating fixed values, probability distributions (i.e., inputs for C_food_ and C_content_) were used for both the food consumption and CPF residue-level data. Monte Carlo simulation, conducted with 10,000 iterations in @RISK within Lumivero’s Desktop Decision Tool, was applied in this study. The method randomly selected numerous pairs of values from the distributions, performed calculations for each pair, and compiled the results (i.e., outputs) into a histogram that represented the distribution of CPF exposure.

## 3. Results

### 3.1. CPF Occurrence in Tomato and Cucumber

The detected CPF residue levels in locally sourced tomatoes and cucumbers are presented in Table 1. CPF residues, with a mean concentration of 0.003 mg/kg, were found in 15% of tomato samples (3 out of 20) and in 28.6% of cucumber samples (4 out of 14). Overall, the detected CPF residue levels ranged from 0.002 mg/kg to 0.005 mg/kg. None of the analyzed samples contained CPF residues exceeding the EU’s former MRL of 0.01 mg/kg, which was applicable prior to the ban on CPF.

Armenia is a member of the Eurasian Economic Union (EAEU), and the technical regulation on food safety applicable within EAEU member countries (Armenia, Kazakhstan, Kyrgyzstan, Belarus, and Russia) establishes MRLs for various contaminants, including pesticides in vegetables [31]. However, it is important to note that no specific MRLs for CPF in vegetables, including tomatoes and cucumbers, are currently set or applicable under this regulation. Another regulatory document [32], which covers unified sanitary-epidemiological and hygienic requirements for food products across EAEU member countries, sets an MRL of 0.05 mg/kg for selected vegetables, such as potatoes, carrots, and cabbage, but does not include other widely consumed vegetables like tomatoes and cucumbers. Hence, although Armenia lacks a nationally established MRL for CPF, the detected residue levels in the studied vegetables (Table 1) were well below the EU’s former MRL of 0.01 mg/kg.

### 3.2. Chronic and Acute Dietary Exposure to CPF

The chronic and acute dietary exposure to CPF through the consumption of locally sourced tomatoes and cucumbers among the adult population of Armenia was assessed based on CPF residue levels and food consumption patterns. Consumers were categorized into five groups based on their individual consumption levels of each vegetable to enable a detailed assessment of CPF exposure, taking into account varying dietary patterns, including low and high consumers.

Table 2 summarizes the chronic estimated daily intake (EDI) of CPF associated with individual and combined consumption of tomatoes and cucumbers by the adult population. For tomato, the mean chronic EDI values for all consumers (n = 1188, excluding 5th percentile of outliers) was 1.26 × 10^−5^ mg/kg bw/day, with a range of 6.35 × 10^−7^ to 3.09 × 10^−5^ mg/kg bw/day.

Among the identified consumer groups, Group 1 (19.19% of tomato consumers, lowest consumption) had the lowest mean EDI of 3.99 × 10^−6^ mg/kg bw/day, while Group 5 (18.52% of tomato consumers, highest consumption) had the highest mean EDI at 2.49 × 10^−5^ mg/kg bw/day. A similar pattern was observed in the case of chronic CPF exposure via cucumber consumption. For cucumber, the mean chronic EDI values for all consumers was lower, at 6.99 × 10^−6^ mg/kg bw/day, with the range of 7.48 × 10^−8^ to 1.82 × 10^−5^ mg/kg bw/day. Group 1 (21.86% of cucumber consumers, lowest consumption) showed a mean EDI of 1.58 × 10^−6^ mg/kg bw/day, while Group 5 (21.44% of cucumber consumers, highest consumption) reached a mean EDI of 1.50 × 10^−5^ mg/kg bw/day. The cumulative chronic exposure (∑EDI) in the case of the consumption of both studied vegetables was calculated at 1.96 × 10^−5^ mg/kg bw/day, ranging from 7.09 × 10^−7^ to 4.91 × 10^−5^ mg/kg bw/day.

While chronic EDI values were calculated based on average daily consumption data, acute EDI values were derived from consumed portions of tomatoes and cucumbers. Consequently, the acute dietary exposure estimates (Table 3) showed a relatively higher mean intake of CPF.

The acute EDI via tomato consumption for all consumers was 1.57 × 10^−5^ mg/kg bw/day, ranging from 3.86 × 10^−6^ to 3.47 × 10^−5^ mg/kg bw/day. Group 5, representing the highest consumers, had a mean acute EDI of 2.57 × 10^−5^ mg/kg bw/day. For cucumber consumption, the mean acute EDI was 8.52 × 10^−6^ mg/kg bw/day, with the highest consumer group (Group 5) having a mean acute daily intake of 1.72 × 10^−5^ mg/kg bw/day. The cumulative mean acute exposure (∑EDI) from both vegetables was estimated at 2.42 × 10^−5^ mg/kg bw/day.

### 3.3. Deterministic and Probabilistic Risk Assessment of CPF

The risk assessment of chronic and acute dietary exposure to CPF through tomato and cucumber consumption was conducted using MOE for chronic exposure and HQ for acute exposure. Table 4 and Table 5 present the results of the deterministic assessments for chronic (MOE) and acute (HQ) risks, respectively.

In the case of tomato consumption, the mean MOE (Table 4) for all consumers was 2.38 × 10^4^, ranging from 9.72 × 10^3^ to 4.73 × 10^5^, while for cucumber consumption, the mean MOE was 4.29 × 10^4^, with a range of 1.65 × 10^4^ to 4.01 × 10^6^. The mean cumulative chronic risk (∑MOE) from both vegetables was 1.53 × 10^4^, with a range of 6.12 × 10^3^ to 4.23 × 10^5^.

**Figure 1:**
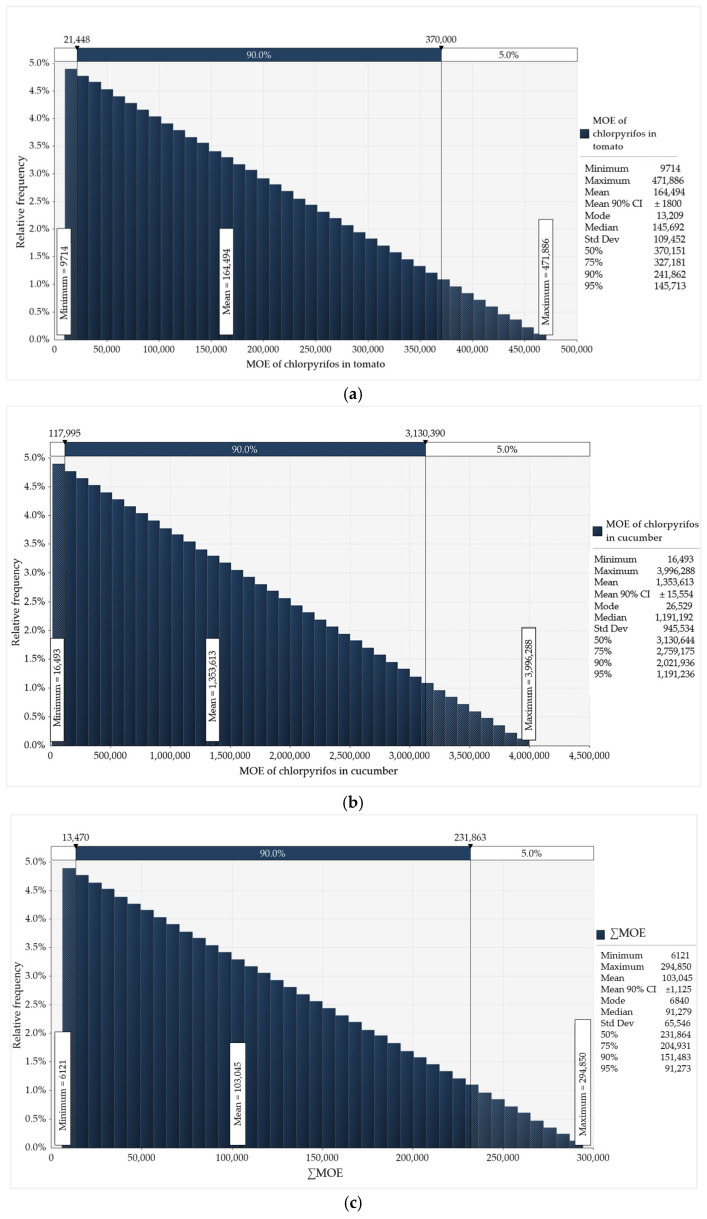
Probabilistic assessment of chronic dietary risk from CPF exposure through consumption of (**a**) tomato and (**b**) cucumber, and (**c**) cumulative chronic risk (∑MOE).

**Figure 2:**
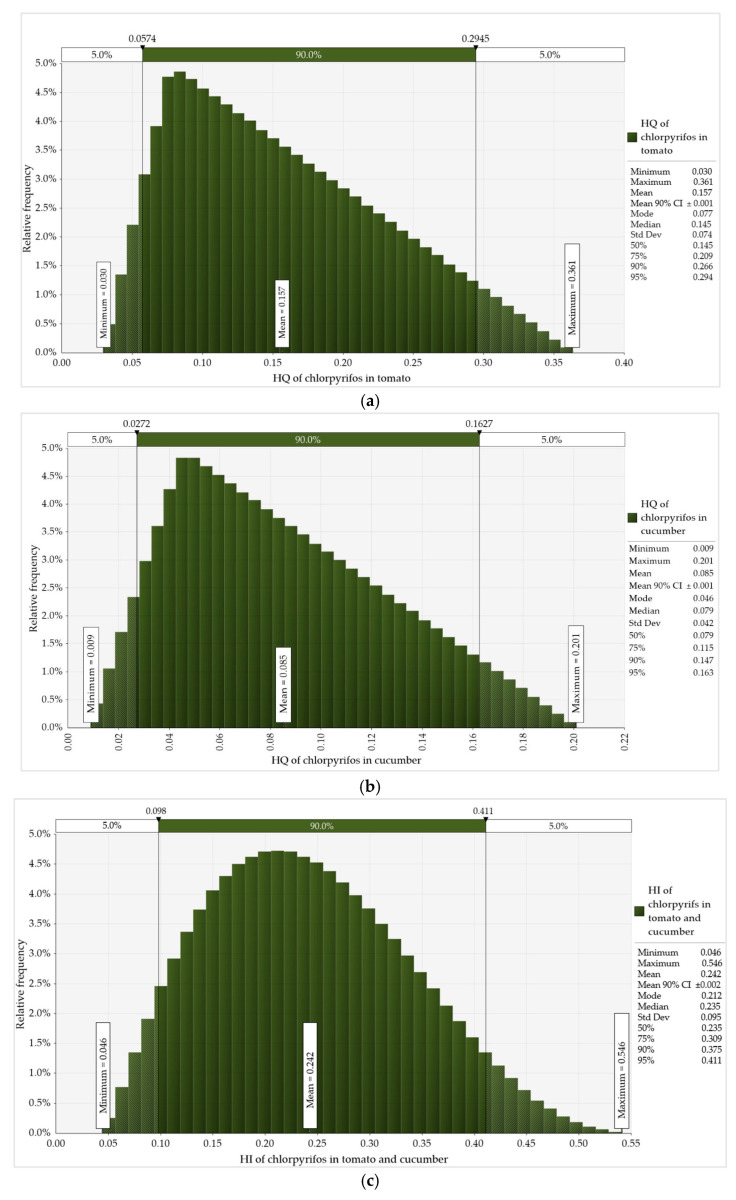
Probabilistic assessment of acute dietary risk from CPF exposure through consumption of (**a**) tomato and (**b**) cucumber, and (**c**) cumulative acute risk (∑HQ = HI).

The mean HQ (Table 5) for tomato and cucumber consumers was 0.16 (range of 0.04–0.35) and 0.09 (range of 0.01–0.18), respectively. The mean cumulative acute risk (∑HQ, i.e., HI) from the combined consumption of the two studied vegetables was 0.24, with a range of 0.05 to 0.53. It is important to note that the HQ and HI data presented in the study are based on the lowest ARfD of 0.0001 mg/kg bw/day. Since the risk values calculated using this more conservative reference value are already very low, the outcomes obtained using the higher ARfD of 0.005 mg/kg bw/day would be even lower. In contrast to the deterministic assessments, which were based solely on point estimates for the general population and identified consumer groups, the probabilistic risk assessment of chronic and acute dietary exposure to CPF from tomato and cucumber consumption incorporated two types of variables: consumption data from all respondents who reported consuming the studied vegetables, and the residue levels detected in the samples. The results of the Monte Carlo simulations for chronic and acute risk assessments, each performed with 10,000 iterations in @RISK within Lumivero’s Desktop Decision Tool, are presented in Figure 1 and Figure 2, respectively.

The figures include statistical parameters such as mean, median, mode, mean 90% confidence interval (CI), percentiles (P50, P75, P90, P95) and the minimum, maximum, and standard deviation (Std Dev). The results of the probabilistic assessments of chronic (Figure 1) and acute (Figure 2) dietary risks from CPF exposure through tomato and cucumber showed distinct distributional patterns, with a wide range of confidence intervals (e.g., 90% CI). In @Risk, the distributions for HQs and MOEs were defined by the tool as Triangular based on the best fit to the input data, while for the ∑HQ (i.e., HI) and ∑MOE, the Pert distribution was applied. In addition, the consumption data followed a Beta General distribution.

The probabilistic risk assessment of CPF chronic exposure revealed lower MOE values for tomato consumption (Figure 1a) compared to cucumber consumption (Figure 2b). The probabilistic estimates for cumulative chronic risk (∑MOE) from CPF exposure through combined consumption of the two studied vegetables resulted in a ∑MOE distribution with a minimum of 6,121 and maximum of 294,850 (Figure 1c).

The acute probabilistic risk assessment for CPF via tomato consumption generated HQ estimates with a minimum of 0.03 and a maximum of 0.361 (Figure 2a). The similar estimates in the case of cucumber consumption were relatively lower as HQ ranged from a minimum of 0.009 to a maximum of 0.201 (Figure 2b). The cumulative acute probabilistic CPF risk, expressed as HI, the sum of HQs (∑HQ = HI), showed higher values with a minimum of 0.046 and a maximum of 0.546, indicating relatively increased estimates compared to individual vegetable consumption scenarios.

## 4. Discussion

### 4.1. CPF Residues in Tomato and Cucumber

Although CPF is a non-authorized pesticide in several countries, it remains permitted in Armenia and is used in agriculture, as evidenced by CPF residues detected in 15% of tomato samples and in 28.6% of cucumber samples in this study. Comparisons with similar cases reported by the Rapid Alert System for Food and Feed (RASFF) portal [22] reveal notifications of unauthorized CPF residues in fresh products, including tomatoes imported from Egypt (0.14 ± 0.070 mg/kg, notified by Cyprus), tomatoes from Turkey (0.055 ± 0.028 mg/kg, notified by Greece; 0.079 mg/kg, notified by Croatia), cucumbers from Serbia (0.060 mg/kg, notified by Croatia), and cucumbers from Poland (0.039 mg/kg, notified by the Czech Republic). Notable, these reported levels exceed those found in locally sourced tomatoes and cucumbers consumed in Armenia.

The occurrence of CPF residues in vegetables, as reported globally, particularly by Tudi et al. [4], the International Pollutants Elimination Network [33], Bitencourt de Morais Valentim et al. [34], and Elguenta et al. [35], points out the challenges related to the control of CPF residues in widely consumed vegetables. Even post-ban, illegal use and residue detections continue [4]. Interestingly, in Armenia, the detected CPF residue levels were lower than those reported in China (0.01–0.15 mg/kg) [4], India (0.02–0.1 mg/kg in tomatoes and other products) [33], Brazil (up to 0.05 mg/kg in tomatoes) [34], and Chile (up to 0.08 mg/kg in tomatoes) [35]. Studies [33] recommend phasing out and imposing stricter regulations on high-risk pesticide, including CPF, due to their cumulative risks, particularly when considering the consumption of common foods such as tomatoes and other vegetables. Consistent with the earlier discussion on the absence of specific MRLs for vegetables such as tomatoes and cucumbers in EAEU regulations, the findings of this study highlight a regulatory gap that may lead to inconsistent safety standards in Armenia. Compliance with food safety requirements emphasizes the need for Armenia and the EAEU to establish crop-specific MRLs to enhance consumer protection, particularly for widely consumed products like tomatoes and cucumbers. Moreover, the focus should be on working toward banning or restricting high-risk pesticides or on identifying alternatives to CPF to replace it with substances that present lower risks and environmental residues. For example, pymetrozine and avermectin are mentioned in the literature [4] as safer alternatives to CPF. In addition, continuous monitoring programs that include a broader variety and larger sample size of plant-origin products, with an emphasis on multiple-pesticide investigations, are crucial.

### 4.2. CPF Dietary Exposure

The CPF dietary exposure estimates, expressed as EDIs, varied across different consumption patterns of tomatoes and cucumbers, highlighting the influence of individual dietary habits of the population. Notably, higher EDIs were obtained for high-consumption groups, particularly Group 4 and, to a greater extent, Group 5 (the highest consumption group), which are characterized by relatively higher consumption rates. It is important to note that these consumer groups were identified based on tomato and cucumber consumption rates rather than demographic characteristics, such as age or gender. Consumers of tomatoes and cucumbers, aged 18 years and older and including both males and females, were represented in each identified group, indicating that CPF dietary exposure is attributed more by individual consumption habits than by age or gender. Similarly to this study, Elguenta et al. also highlighted the role of local dietary patterns and regulatory approaches when considering the variations in the daily intake of CPF within different regions, while reporting that studies conducted in Chile found that pesticides, including CPF, reached relatively high daily intake values in the population [35].

Although CPF has been banned and the EFSA currently does not establish or recommend an ADI for it, comparing the results of this study with the previously established ADI of 0.001 mg/kg bw/day remains informative. This ADI value was reduced several times before the ban, reflecting growing concerns about CPF exposure [13,14]. All EDI values obtained in this study were well below this former guidance value (0.001 mg/kg bw/day), indicating low levels of CPF dietary exposure through tomato and cucumber consumption among the studied adult population. It is relevant to mention that the exposure estimates do not reflect lifetime CPF exposure, as the EDI calculations do not account for exposure duration (considering population life expectancy) or exposure frequency.

### 4.3. CPF Risk to Consumers of Tomato and Cucumber

Both deterministic and probabilistic assessments of chronic and acute risk indicated no health concerns associated with CPF exposure due to tomato and cucumber consumption. The use of Monte Carlo simulation effectively captured exposure variability, highlighting the advantage of a probabilistic approach in evaluating both typical and high consumption scenarios, thus providing a comprehensive framework for CPF-related dietary risk assessment.

Chronic dietary risk, assessed using MOE, was based on the DNT LOAEL of 0.3 mg/kg bw/day [15] and compared with a concern level of 300. All deterministic and probabilistic MOE estimates were well above the health concern level (MOE > 300 and >1000 [14]), indicating no chronic health risks for the studied population from the consumption of locally sourced tomatoes and cucumbers. For instance, even the lowest MOE (∑MOE of 4.23 × 10^3^) observed among high consumers (Group 5) was still above the threshold. For high consumers, represented by the P95 (95th percentile) value from the Monte Carlo Simulation, the ∑MOE was 91,273, which is well above the health concern level, indicating no health concern even in this subgroup. Acute dietary risk was assessed using HQ and, for cumulative risk from the combined consumption of both studied vegetables, the HI, based on an artificially low ARfD of 0.0001 mg/kg bw/day [30]. All deterministic and probabilistic HQ and HI values among consumer groups remained well below the threshold of 1 (HQ and HI < 1), indicating no acute health concern. Notably, the cumulative acute risk (HI) from the combined consumption of both vegetables, as estimated through deterministic assessment, amounted to 24% of the threshold value of 1. Among high consumers, the contribution of both vegetables reached up to 53% in deterministic assessments and 54.6% in probabilistic assessments, indicating that high consumption of these two vegetables alone accounts for more than half of the safety threshold for CPF acute exposure. When mentioning cumulative risk, it is noteworthy that this study conducted in Armenia focused on only one pesticide; however, potential cumulative exposure to multiple pesticides with similar mechanism of action should be considered, as it may pose a higher risk [36,37,38].

Interestingly, similar studies on CPF risk assessment through tomato and cucumber consumption in neighboring countries are scarce. For example, a study conducted in Turkey reported no reason for concern regarding cumulative exposure to pesticides, including CPF, in cucumbers for the Turkish population [39]. Another study from Iran on greenhouse-grown tomatoes found HQ values below 1. The Monte Carlo simulation in this Iranian study indicated that HQ values were more influenced by consumption rate, followed by pesticide concentration and body weight [40]. Similarly, the sensitivity analysis conducted in @Risk, aiming to identify which input variables most significantly influence the risk outcomes for CPF in Armenia, showed that risk values (i.e., MOE, HQ, HI) were most attributed by tomato and cucumber consumption rates.

A global systematic [41] review with meta-analysis indicated that for the non-carcinogenic health risk assessments conducted using HQ estimates, there were no health concerns associated with the exposure to organophosphorus pesticides that included CPF from vegetable consumption: the HQ values were always below 1 (i.e., HQ < 1).

## 5. Conclusions

This study is the first to assess CPF residues in locally sourced tomatoes and cucumbers in Armenia, addressing a critical gap in national food safety data and providing the baseline information for the country, while highlighting the need for continuously expanding monitoring programs. By focusing on two of the most commonly consumed vegetables and applying both deterministic and probabilistic approaches for assessments, this study provides a strong starting point for evaluating pesticide exposure risks for the Armenian population. Internationally, the findings contribute to a broader understanding of CPF occurrence in commonly consumed vegetables and exposure in countries where regulatory restrictions on CPF may be recently adopted or poorly enforced. Nationally, the study provides essential evidence to support science-based decision-making by food safety authorities, and to guide risk management strategies aligned with international practices, particularly in the absence of specific MRLs for CPF in tomatoes and cucumbers within EAEU regulations applicable in Armenia.

Both deterministic and probabilistic dietary risk assessments indicate that CPF exposure through tomato and cucumber consumption poses no health concern for the general adult population in Armenia under current residue levels. However, risk values (HI and MOE), particularly for high-consumption groups (e.g., Groups 4 and 5), suggest that these two vegetables alone may notably contribute to overall CPF exposure. The study also indicates the importance of probabilistic modeling in dietary risk assessment, as it accounts for variability in consumption and residue levels, providing understanding on how risk management strategies may increase or decrease CPF exposure.

Dietary exposure estimates in this study were based on CPF residues in fresh tomatoes and cucumbers and did not account for the potential reduction in residues due to common food preparation practices such as washing, peeling, or cooking. Although tomatoes and cucumbers are important contributors to dietary exposure of CPF, other potential dietary exposure sources were not included in this study, limiting its scope. Therefore, future research should expand to include a broader variety of plant-origin products, particularly those included in the national residue monitoring program and with high consumption rates. The additional limitation of this research is that it did not consider specific groups of population, such as children and pregnant women, who are particularly vulnerable to CPF exposure.

Overall, the study outcomes pointed out that there is a need for Armenia to establish crop-specific MRLs for widely consumed vegetables such as tomatoes and cucumbers. It provides scientific basis for Armenia to propose regulatory measures to the EAEU, particularly for setting the specific MRLs. In line with global changes in CPF regulations and trends toward banning or restricting high-risk pesticides in food products, the use of CPF in Armenia emphasizes the need to establish regulatory levels, enhance monitoring, and promote safer agricultural practices for alternative pest management at an international level.

## Figures and Tables

**Table 1:** CPF residue levels (mg/kg) in studied vegetables.

CPF Residue Levels (mg/kg)	Studied Vegetables	
	Tomato	Cucumber
% of N/D samples	85.0%	71.4%
Mean ± SD	0.003 ± 0	0.003 ± 0.001
Minimum	0.003	0.002
Maximum	0.003	0.005

Note: N/D—non-detected; SD—standard deviation.

**Table 2:** Chronic EDI of CPF via consumption of tomato and cucumber.

Studied Vegetables	Identified Groups of Consumers	Percentage (%) of Consumers in Each Group	Chronic EDI of CPF (mg/kg bw/day)	
			Mean EDI	EDI Range (Min–Max)
Tomato	All consumers	100 (1188 respondents)	1.26 × 10^−5^	6.35 × 10^−7^–3.09 × 10^−5^
	Group 1	19.19	3.99 × 10^−6^	≤6.60 × 10^−6^
	Group 2	23.23	7.71 × 10^−6^	7.10 × 10^−6^–7.72 × 10^−6^
	Group 3	32.24	1.37 × 10^−5^	7.87 × 10^−6^–1.54 × 10^−5^
	Group 4	6.82	1.89 × 10^−5^	1.65 × 10^−5^–1.93 × 10^−5^
	Group 5	18.52	2.49 × 10^−5^	≥1.98 × 10^−5^
Cucumber	All consumers	100 (1194 respondents)	6.99 × 10^−6^	7.48 × 10^−8^–1.82 × 10^−5^
	Group 1	21.86	1.58 × 10^−6^	≤2.92 × 10^−6^
	Group 2	8.54	4.33 × 10^−6^	3.21 × 10^−6^–4.55 × 10^−6^
	Group 3	29.65	6.54 × 10^−6^	4.86 × 10^−6^–8.94 × 10^−6^
	Group 4	18.51	9.49 × 10^−6^	8.96 × 10^−6^–1.14 × 10^−5^
	Group 5	21.44	1.50 × 10^−5^	≥1.17 × 10^−5^
Cumulative chronic exposure (∑EDI)			1.96 × 10^−5^	7.09 × 10^−7^–4.91 × 10^−5^

**Table 3:** Acute EDI of CPF via consumption of tomato and cucumber.

Studied Vegetables	Identified Groups of Consumers	Percentage (%) of Consumers in Each Group	Acute EDI of CPF (mg/kg bw/day)	
			Mean EDI	EDI Range (Min–Max)
Tomato	All consumers	100 (1181 respondents)	1.57 × 10^−5^	3.86 × 10^−6^–3.47 × 10^−5^
	Group 1	19.19	7.18 × 10^−6^	≤7.72 × 10^−6^
	Group 2	23.23	1.12 × 10^−5^	7.87 × 10^−6^–1.16 × 10^−5^
	Group 3	32.24	1.54 × 10^−5^	1.23 × 10^−5^–1.54 × 10^−5^
	Group 4	6.82	1.92 × 10^−5^	1.70 × 10^−5^–1.93 × 10^−5^
	Group 5	18.52	2.57 × 10^−5^	≥2.08 × 10^−5^
Cucumber	All consumers	100 (1172 respondents)	8.52 × 10^−6^	1.14 × 10^−6^–1.82 × 10^−5^
	Group 1	21.86	4.09 × 10^−6^	≤4.55 × 10^−6^
	Group 2	8.54	6.80 × 10^−6^	5.68 × 10^−6^–6.82 × 10^−6^
	Group 3	29.65	9.09 × 10^−6^	8.18 × 10^−6^–9.09 × 10^−6^
	Group 4	18.51	1.30 × 10^−5^	1.05 × 10^−5^–1.36 × 10^−5^
	Group 5	21.44	1.72 × 10^−5^	≥1.59 × 10^−5^
Cumulative acute exposure (∑EDI)			2.42 × 10^−5^	5.00 × 10^−6^–5.29 × 10^−5^

**Table 4:** Deterministic assessment of chronic risk from CPF through consumption of tomato and cucumber.

Studied Vegetables	Identified Groups of Consumers	Margin of Exposure (MOE) of CPF	
		Mean MOE	MOE Range (Min–Max)
Tomato	All consumers	2.38 × 10^4^	9.72 × 10^3^–4.73 × 10^5^
	Group 1	7.52 × 10^4^	≤4.55 × 10^4^
	Group 2	3.89 × 10^4^	3.89 × 10^4^–4.23 × 10^4^
	Group 3	2.18 × 10^4^	1.94 × 10^4^–3.81 × 10^4^
	Group 4	1.59 × 10^4^	1.55 × 10^4^–1.82 × 10^4^
	Group 5	1.21 × 10^4^	≥1.52 × 10^3^
Cucumber	All consumers	4.29 × 10^4^	4.01 × 10^4^–1.65 × 10^6^
	Group 1	1.90 × 10^5^	≤1.03 × 10^5^
	Group 2	6.92 × 10^4^	6.60 × 10^4^–9.33 × 10^4^
	Group 3	4.59 × 10^4^	3.35 × 10^4^–6.17 × 10^4^
	Group 4	3.16 × 10^4^	2.64 × 10^4^–3.35 × 10^4^
	Group 5	2.00 × 10^4^	≥ 2.57 × 10^4^
Cumulative chronic risk (∑MOE)		1.53 × 10^4^	4.23 × 10^3^–6.12 × 10^5^

**Table 5:** Deterministic assessment of acute risk from CPF through consumption of tomato and cucumber.

Studied Vegetables	Identified Groups of Consumers	Hazard Quotient (HQ) of CPF	
		Mean HQ	HQ Range (Min–Max)
Tomato	All consumers	0.16	0.04–0.35
	Group 1	0.07	≤0.08
	Group 2	0.11	0.08–0.12
	Group 3	0.15	0.12–0.15
	Group 4	0.19	0.17–0.19
	Group 5	0.26	≥0.21
Cucumber	All consumers	0.09	0.01–0.18
	Group 1	0.04	≤0.05
	Group 2	0.07	0.06–0.07
	Group 3	0.09	0.08–0.09
	Group 4	0.13	0.10–0.14
	Group 5	0.17	≥0.16
Cumulative acute risk (∑HQ = HI)		0.24	0.05–0.53

## Data Availability

The original contributions presented in this study are included in the article. Further inquiries can be directed to the corresponding author.

## Abbreviations

The following abbreviations are used in this manuscript:

ADI	Acceptable Daily Intake;
ARfD	Acute Reference Dose;
BW	Body Weight;
CENS	Center for Ecological-Noosphere Studies;
CI	Confidence Interval;
CPF	Chlorpyrifos;
DNT	Developmental Neurotoxicity;
EAEU	Eurasian Economic Union;
EC	European Commission;
ED	Endocrine Disruption;
EDI	Estimated Daily Intake;
EFSA	European Food Safety Authority;
HBGV	Health-Based Guidance Value;
HI	Hazard Index;
HQ	Hazard Quotient;
IPCS	International Programme on Chemical Safety;
LOAEL	Lowest Observed Adverse Effect Level;
MOE	Margin of Exposure;
MRL	Maximum Residue Limit;
RASFF	Rapid Alert System for Food and Feed;
SOPs	Standard Operational Procedures;
US EPA	United States Environmental Protection Agency;
WHO	World Health Organization.
